# Feature Importance Analysis of a Deep Learning Model for Predicting Late Bladder Toxicity Occurrence in Uterine Cervical Cancer Patients

**DOI:** 10.3390/cancers15133463

**Published:** 2023-07-02

**Authors:** Wonjoong Cheon, Mira Han, Seonghoon Jeong, Eun Sang Oh, Sung Uk Lee, Se Byeong Lee, Dongho Shin, Young Kyung Lim, Jong Hwi Jeong, Haksoo Kim, Joo Young Kim

**Affiliations:** 1 Proton Therapy Center, National Cancer Center, Goyang-si 10408, Republic of Korea; wonjoongcheon@gmail.com (W.C.);; 2 Biostatistics Collaboration Team, National Cancer Center, Goyang-si 10408, Republic of Korea

**Keywords:** uterine cervical cancer, toxicity prediction, deep learning, feature importance, interpretable artificial intelligence

## Abstract

**Simple Summary:**

This study developed a prediction model for late bladder toxicity in patients with uterine cervical cancer undergoing radiation therapy. A deep learning (DL) model was trained on data from 281 patients and compared its performance with a multivariable logistic regression model. The DL model outperformed the regression model, achieving higher accuracy, recall, F1-score, and area under the receiver operating characteristic curve. Specifically, based on the feature importance analysis, the DL model identified the doses for the most exposed 2 cc volume of the bladder (BD_2cc_), BD_5cc_, and ICRU bladder point as high-priority features. Finally, the lightweight DL model, which was designed to focus on the top five important features, demonstrated superior predictive capabilities, highlighting its potential in improving patient outcomes and minimizing treatment-related complications with secured reliability.

**Abstract:**

(1) In this study, we developed a deep learning (DL) model that can be used to predict late bladder toxicity. (2) We collected data obtained from 281 uterine cervical cancer patients who underwent definitive radiation therapy. The DL model was trained using 16 features, including patient, tumor, treatment, and dose parameters, and its performance was compared with that of a multivariable logistic regression model using the following metrics: accuracy, prediction, recall, F1-score, and area under the receiver operating characteristic curve (AUROC). In addition, permutation feature importance was calculated to interpret the DL model for each feature, and the lightweight DL model was designed to focus on the top five important features. (3) The DL model outperformed the multivariable logistic regression model on our dataset. It achieved an F1-score of 0.76 and an AUROC of 0.81, while the corresponding values for the multivariable logistic regression were 0.14 and 0.43, respectively. The DL model identified the doses for the most exposed 2 cc volume of the bladder (BD_2cc_) as the most important feature, followed by BD_5cc_ and the ICRU bladder point. In the case of the lightweight DL model, the F-score and AUROC were 0.90 and 0.91, respectively. (4) The DL models exhibited superior performance in predicting late bladder toxicity compared with the statistical method. Through the interpretation of the model, it further emphasized its potential for improving patient outcomes and minimizing treatment-related complications with a high level of reliability.

## 1. Introduction

Cervical cancer is the third most commonly diagnosed malignancy in women worldwide, as reported in 2022 [1]. With early detection and advances in treatment, the number of cervical cancer survivors has increased over the past 40 years. Nevertheless, most patients with cervical cancer suffer from various morbidities resulting from the disease itself or from the treatment. Depending on the treatment strategy (surgery, radiotherapy (RT), or chemotherapy), the morbidities may include symptoms associated with the gastrointestinal or urinary tract, lymphedema, and sexual dysfunctions.

External beam radiation therapy (EBRT) with concurrent chemotherapy followed by intracavitary brachytherapy represents the standard treatment for locally advanced cervical cancers [2,3]. Owing to the very high dose of radiation delivered to the pelvic area using EBRT and brachytherapy, radiation-induced toxicity in these patients is not negligible [4]. Among the irradiated pelvic organs, radiation-induced bladder toxicity is the most commonly observed morbidity in cervical cancer patients who have received curative radiotherapy, with an incidence of approximately 20% [4]. Although severe bladder toxicities have become rare with image-guided brachytherapy, they still pose a considerable risk [5].

Predicting the occurrence of bladder toxicity before treatment is crucial for improving patients’ long-term health outcomes and quality of life, as it reduces the probability of treatment-related complications or interruptions. Previous studies have focused on investigating prognostic factors associated with radiation-induced bladder toxicity. These studies have examined various dose volumetric parameters, such as the doses for the most exposed 2 cc volume of the bladder (BD_2cc_) [5] and the volume receiving a certain biologically weighted equivalent dose (EQD2) relative to the gross tumor volume (GTV) or clinical target volume (CTV), such as V_51.43Gy_ [6] and V_8.5Gy/w_ [7].

Statistical methods and deep learning (DL) models have been introduced to predict the toxic effects of radiation therapy on the gastrointestinal [8,9,10,11,12,13,14,15] and genitourinary systems [6,7,16,17,18,19,20,21,22,23,24]. Statistical methods such as univariable or multivariable linear regression, logistic regression [6,7,10,13,16,17,19], Cox regression [16,17,19,20,22], random forest [11], support vector machine [15,21,24], genetic algorithms [21], and statistical analysis [8,9,12] have been used to predict clinical outcomes. In DL methods, convolutional neural networks (CNN) and multilayer perceptrons (MLP) have been employed to predict radiation-induced toxicity [14,21].

Related studies that have used predictive models for toxicity in various cancer types and radiation therapy techniques:

- A machine-learning-based prediction model of fistula formation after interstitial brachytherapy for locally advanced gynecological malignancies achieved an accuracy of 0.901 using Support Vector Machine (SVM) [24].
- A feasibility study utilized a deep convolutional neural network (CNN) with transfer learning to predict rectum toxicity in cervical cancer radiotherapy, achieving an AUC of 0.89 [14].
- An observational study predicting radiotherapy impact on late bladder toxicity in prostate cancer patients used univariate logistic regression, achieving an AUC of 0.626 [6].
- Various studies have focused on predicting urinary toxicity in prostate cancer radiotherapy using different models, such as the international prostate symptoms score model, logistic and Cox regression, the edited nearest neighbor algorithm together with the regularized discriminant analysis classifier, and others [16,18,19].
- Predicting late organ-at-risk toxicity after prostate radiation therapy has been explored using statistical analysis, cox regression, and random forest models [11,22,23].
- In radiotherapy for cervical cancer, radiomics analysis of 3D dose distributions has been employed to predict toxicity rates, achieving AUCs ranging from 0.57 to 0.89 [13].

The differences between the statistical and DL methods can be described based on three factors: (i) model complexity, (ii) feature importance, and (iii) model transparency. Statistical methods tend to exhibit relatively low complexity, and, compared with DL models, they operate based on clear governing principles. Statistical methods often demonstrate relatively low accuracy; however, they offer relatively high model transparency, which simplifies the interpretation of feature importance. DL models tend to outperform statistical methods. However, they are more complex [25,26,27] and considered “black boxes”, which makes the interpretation of their results very challenging [28]. However, both accuracy and interpretability must be considered when choosing a method for predicting clinical outcomes [29,30,31,32].

Therefore, in the present study, we propose an interpretable DL model for predicting late bladder toxicity in patients with cervical cancer who have received definitive radiotherapy. We compared the performance of the statistical method and the DL model. In addition to achieving a high level of reliability, we conducted a feature-importance analysis and validated the performance of a lightweight DL model.

## 2. Materials and Methods

### 2.1. Patient Selection

We identified 545 patients with primary uterine cervical cancer who underwent definitive RT with curative intent at our institution between February 2006 and December 2017. Follow-up evaluations were performed every three months in the first two years, every four months in the third year, every six months in the fourth and fifth years, and annually thereafter. In this study, we included patients with more than three years of follow-up after treatment completion. The radiation-induced bladder toxicity was evaluated during the regular follow-up visits based on the European Organization for Research and Treatment of Cancer late radiation toxicity criteria, which represent a structured scoring schema developed by the Radiation Therapy Oncology Group (RTOG) [33].

**Figure 1:**
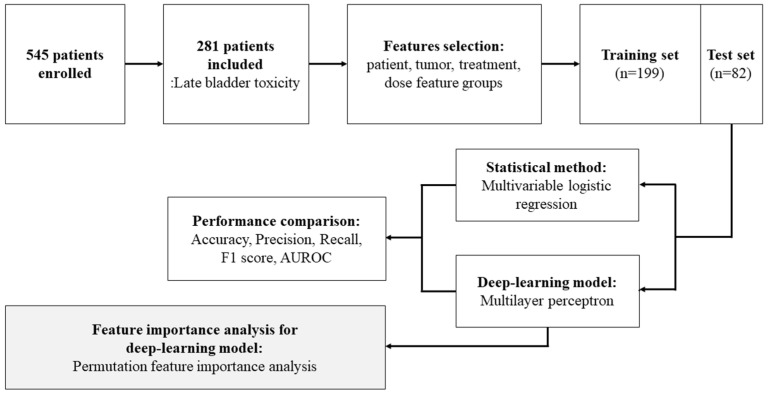
Flowchart of the proposed method for late bladder toxicity occurrence prediction using a deep learning model and feature importance analysis.

In total, 281 patients with cervical cancer were included in this study (Figure 1). Clinical information regarding the status of the disease and treatment-related complications was collected retrospectively from patients’ medical records. This study was approved by the Institutional Review Board (IRB) of the National Cancer Center, Korea: NCC2019-0166. This study adheres to the tenets of the Helsinki Declaration of 1975. The requirement for informed consent was waived owing to the retrospective nature of the study. 

### 2.2. Treatment

The patients received concurrent chemoradiotherapy using either three-dimensional (3D) conformal EBRT or an intensity-modulated RT technique and high-dose-rate brachytherapy. The clinical target volume of EBRT included gross disease, the entire uterus, a margin of 2.0 cm around the tumor extent at the level of the vagina, and the entire parametrial tissue. In addition, regional lymphatics, including the common, internal, and external iliac nodal regions and the presacral and para-aortic lymph nodes, were considered. Prior to 2008, brachytherapy planning was CT-based; afterwards, MRI-based planning has been employed [4]. Brachytherapy planning generally followed the recommendations of the Gynecologic Group European de Curie Therapie, European Society for Therapeutic Radiology, and Oncology Working Group [34,35].

The patients received a combination of 45–50 Gy of pelvic EBRT and 30 Gy of high-dose-rate brachytherapy with daily fractions of 5 Gy over 3 weeks. The biologically equivalent dose to point A in 2 Gy fractions was previously calculated as approximately 82 Gy for cervical tumors (α/β = 10) and 91 Gy for normal tissue (α/β = 3) [36]. Concomitant chemotherapy was administered using a weekly schedule of intravenous cisplatin at 40 mg per square meter of body surface area.

### 2.3. Data

We used four groups of the feature set as input data for the statistical analysis and DL method: (i) “patient” feature group (n = 2), including age and pathology; (ii) “tumor” feature group (n = 3), including the federation of gynecology and obstetrics (FIGO) stage, tumor-node-metastasis (TNM) category, and maximum tumor length on axial T2-weighted magnetic resonance image (MRI); (iii) “treatment” feature group (n = 4), including concurrent chemoradiotherapy (CCRT), CCRT regimen, number of CCRT cycles, and adjuvant chemotherapy; (iv) “dose” feature group with the equieffective dose (EQD2) at 3.0 Gy (n = 7), including the total dose of external beam radiation therapy (EBRT)_,_ the dose delivered to 100% of the primary GTV (GTV-D100), International Commission on Radiation Units and Measurements (ICRU) bladder point (BP_ICRU_), BD_0.1cc_, BD_1.0cc_, BD_2.0cc_, and BD_5.0cc_. Features that were not continuous were dummy-coded. All features were deliberately collected in accordance with IRB guidelines and based on clinical experience and relevant literature [5,36,37,38,39]. These carefully selected features are known to play a significant role in influencing local control and survival outcomes in cervical cancer. With these selections, we aimed to facilitate meaningful comparisons with other studies that have similar diseases. 

Late bladder toxicity was graded according to the scoring schema of the RTOG, with scores in the range of 0–5. The median durations from the initial start of EBRT to the occurrence of late toxicity are 37.7 months in the current study population. Owing to the imbalance of grades, the grade of late bladder toxicity was binarized by considering the occurrence of late bladder toxicity only. When late bladder toxicity is absent, the occurrence status is set to 0; otherwise, the occurrence status is set to 1. 

### 2.4. Statistical Method

Patient characteristics were indicated in terms of mean ± standard deviation or median (min–max) for continuous variables; frequencies and percentage values were used for categorical variables. Continuous variables were compared using the *t*-test or Wilcoxon rank-sum test, whereas categorical variables were compared using the Chi-square or Fisher’s exact tests. The dataset was randomly divided into two sets containing 199 and 82 cases for training and testing the statistical model, respectively. The ratios for the training and test sets were 70% and 30%, respectively. A statistical model was built using a training set with univariable and multivariable logistic regression. A univariable logistic regression was performed on the input data, considering patient, tumor, treatment, and dose features. The results of the univariable logistic regression were used as the input for the multivariable logistic regression. The target was the occurrence of binarized late toxicity. The developed multivariable logistic regression model was tested using the test dataset. Statistical analyses were performed using SAS version 9.4 (SAS Institute Inc., Cary, NC, USA) and R version 4.2.1 (R Foundation for Statistical Computing, Vienna, Austria). We assumed that statistical significance is achieved for *p*-values below 0.05.

### 2.5. Deep Learning Model for Permutation Feature Analysis

To implement the DL model by using Pytorch version 1.12.1 (Warsaw, Mazowieckie, Poland), we used an MLP. MLP is a type of artificial neural network that consists of multiple layers of nodes with batch normalization and nonlinearity functions such as rectified linear unit (RELU), leaky RELU, and sigmoid. Each node in one layer is connected to all nodes in the next layer, forming a fully connected network. The input layer takes the input data, and the output layer produces the final predictions. The layers between the input and output layers are called hidden layers. The DL model was built using six hidden layers. The architecture can be represented mathematically as follows:

(1) $$h_{1}=f_{1}\left(W_{1}x+b_{1}\right),$$

(2) $$h_{2}=f_{2}\left(W_{2}h_{1}+b_{2}\right),$$

(3) $$h_{3}=f_{3}\left(W_{3}h_{2}+b_{3}\right),$$

(4) $$h_{4}=f_{4}\left(W_{4}h_{3}+b_{4}\right),$$

(5) $$h_{5}=f_{5}\left(W_{5}h_{4}+b_{5}\right),$$

(6) $$h_{6}=f_{6}\left(W_{6}h_{5}+b_{6}\right),$$

and

(7) $$y=f_{7}\left(W_{7}h_{6}+b_{7}\right),$$

where *x* is the input data, *y* is the output data, *W_i_* and *b_i_* are the weight matrix and bias vector for the *i*-th layer, respectively, *f_i_* is the activation function for the *i*-th layer, and *h_i_* is the output of the *i*-th hidden layer.

As a preprocessing step, 16 features were used as input data, and binarized late bladder toxicity values were considered the output data. Z-score normalization was applied to the input data so that the DL model could rapidly converge to the optimal solution. The normalization parameters (mean and standard deviation) were determined from the training set.

The standardized input data were passed through an input layer, and output data were obtained on the output layer. In each hidden layer, except for the last one, batch normalization and dropout techniques were applied to avoid overfitting the training data. The probability of a node being zeroed for dropout was set to 0.2. In addition, a leaky ReLU was adopted as the activation function in the hidden layers to improve model complexity and performance. A sigmoid function was used as the activation function of the last layer only. These functions introduce nonlinearity into the model, allowing it to capture complex relationships within the data. The detailed architecture of the proposed model is presented in Table 1. 

The loss between the ground truth and predicted toxicity occurrence was calculated using binary cross-entropy as follows:

(8) $$Binary\;cross\;entropy\;loss=-\frac{1}{N}\sum_{i=1}^{N}\left(y_{i}\log\left(p_{i}\right)+\left(1-y_{i}\right)\log\left(1-p_{i}\right)\right),$$

where *N* represents the total number of samples in the batch, *y_i_* represents the true label of the *i*-th sample (either 0 or 1), and *p_i_* represents the predicted probability (between 0 and 1) of the *i*-th sample belonging to the positive class. The DL models were trained to minimize the loss.

To avoid overfitting, the synthetic minority oversampling technique (SMOTE) was adopted during the training procedure as oversampling technique and augmentation strategy, which adds random noise to the input data in every training epoch [18]. Moreover, we employed an adaptive momentum estimation optimizer with a learning rate of 0.005 and a weight decay of 0.0001. To compute the running average of the gradient, β1 and β2 were set to 0.9 and 0.999, respectively [40]. 

K-fold cross-validation (CV) was employed to ensure the generalizability of the DL model. The value of k was set to 5, resulting in the training set being divided into five folds. Furthermore, an early stopping strategy was implemented, where the model achieving the lowest validation loss during the 500 epochs was saved as the best-performing model. Each fold yielded independent results as separate models were trained. The final output data were determined using the voting method, with a threshold of 3 sets to obtain the ultimate decision.

### 2.6. Permutation Feature Importance Analysis

The permutation analysis is used to calculate the feature importance of a DL model. It involves randomly permuting the value of a single feature and evaluating the model’s performance on the test dataset [41]. The analysis was performed using Scikit-learn version 1.0.2. 

To measure the importance of each feature, the mean squared error (MSE) is calculated between the reference dataset and a corrupted dataset, where the value of a single feature has been randomly permuted. This is compared with the reference MSE, which is the MSE calculated on the uncorrupted reference dataset. 

The variation in model performance resulting from the permutation of a feature is used to compute the importance of each feature.

(9) $$i_{j}=s-\frac{1}{K}\sum_{k=1}^{K}s_{k,j}$$

where *i* is used to index the features, *j* represents the total number of features, $i_{j}$ is the importance of *j*-th feature, *K* is the number of repetitions or permutations, *S* is the reference MSE, $s_{k,j}$ is the MSE calculated using a corrupted dataset for the *k*-th repetition, and $\frac{1}{K}\overset{K}{\underset{k=1}{\sum }}s_{k,j}$ the average MSE over *K* repetitions for *j*-th feature. 

The value of K was set to 500, indicating that the permutation analysis was repeated 500 times. Finally, the permutation feature importance was calculated by averaging the feature importance values obtained from DL models trained using different training folds.

### 2.7. Lightweight Deep Learning Model

In order to develop a lightweight deep learning model, we utilized the importance values derived from permutation feature analysis and selected the top 5 features as input. This approach enabled us to focus on the most influential features while reducing the computational complexity of the model.

When designing the hidden layers, we adhered to the commonly employed standards of multi-layer perceptron (MLP) models. Specifically, our model was designed with a decreasing number of neurons in each hidden layer, starting from the number of input features and gradually reducing to 5, 4, 3, 2, and finally 1 neuron.

The activation functions, preprocessing steps, loss function, optimization technique, cross-validation, and final decision-making process of the lightweight deep learning model remain the same as those of the deep learning model described previously.

### 2.8. Performance Comparison

To compare the performance of the DL model and multivariable logistic regression, four metrics were adopted: accuracy (Equation (10)), precision (Equation (11)), recall (Equation (12)), F1-score (Equation (13)), and the area under the receiver operating characteristic curve (AUROC) (Equation (14)). To compute these metrics, we counted the number of true positives (TP), true negatives (TN), false positives (FP), and false negatives (FN) in the test set. Precision is defined as the number of positive predictions (i.e., those that were correctly identified by the model) expressed as a fraction of the total number of predictions. Recall indicates the fraction of positive instances that the model could identify. The F1-score is the harmonic mean of precision and recall. Therefore, a high F1-score indicates that the model exhibits a good balance between precision and recall. If a model performs well, it has a high AUROC value (close to 1.0).

(10) $$\mathrm{Accuracy}=\frac{\mathrm{TP}+\mathrm{TN}}{\mathrm{TP}+\mathrm{TN}+\mathrm{FP}+\mathrm{FN}}$$

(11) $$\mathrm{Precision}=\frac{\mathrm{TP}}{\mathrm{TP}+\mathrm{FP}}$$

(12) $$\mathrm{Recall}=\frac{\mathrm{TP}}{\mathrm{TP}+\mathrm{FN}}$$

(13) $$F1-\mathrm{score}=2\cdot \frac{\mathrm{Precision}\cdot \mathrm{Recall}}{\mathrm{Precision}+\mathrm{Recall}}$$

(14) $$\mathrm{AUROC}=\int_{0}^{1}\mathrm{sensitivity}\left(x\right)\cdot d\left(1-\mathrm{specificity}\left(x\right)\right)$$

## 3. Results

### 3.1. General Study Characteristics

Patients’ characteristics are summarized in Table 2. No significant differences were observed between the training and test sets in terms of baseline characteristics, such as age, pathology, FIGO stage, TNM category, maximum tumor length, CCRT, CCRT regimen, number of CCRT cycles, adjuvant chemotherapy, and the total dose of EBRT, GTV-D100, BP_ICRU_, BD_0.1cc_, BD_1.0cc_, BD_2.0cc_, and BD_5.0cc_. 

### 3.2. Logistic Regression Results

Univariable and multivariable logistic regression analyses were performed to identify risk predictors for adverse events in the training set (Table 3). Based on the univariable analysis, no significant features were observed in the features set. The multivariable logistic regression model returned four features with *p* < 0.2: CCRT cycle, GTV-D100, BD_2.0cc_, and BD_5.0cc_. The prediction metrics were calculated using a multivariable logistic regression model on the test data. Multivariable logistic regression achieved accuracy, precision, recall, F1-score, and AUROC of 0.85, 0.08, 0.5, 0.14, and 0.43, respectively. Although multivariable logistic regression yielded an accuracy of 0.85, the regression model did not exhibit acceptable performance on the test dataset. 

### 3.3. Permutation Feature Importance: Permutation Analysis

Permutation feature analysis determines the importance of features by quantifying the extent to which the performance of a trained model changes when the dataset is permuted (Equation (7)).

**Figure 2:**
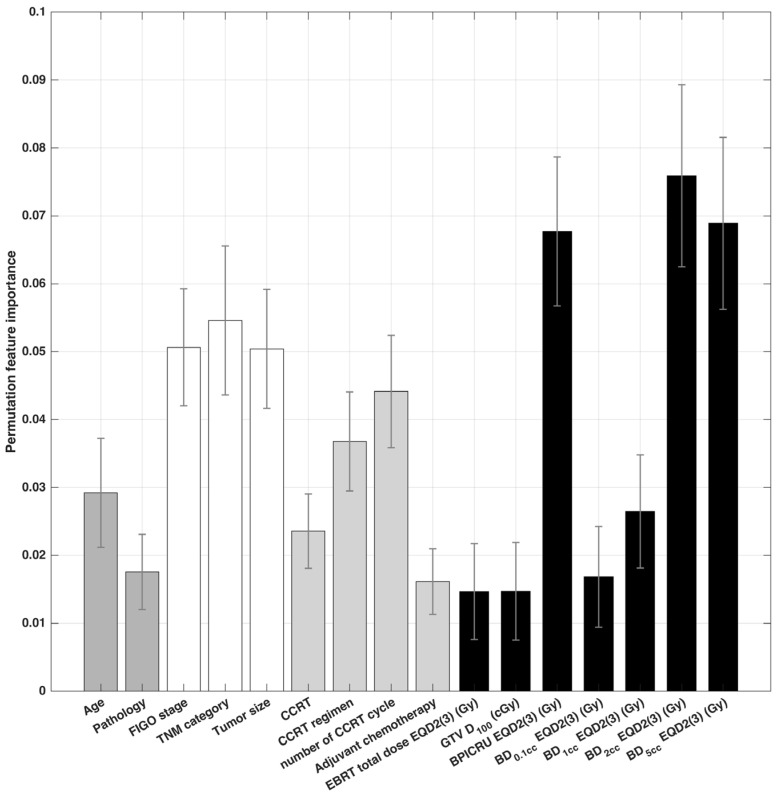
Permutation feature importance computed from the deep learning models.

An independent permutation feature importance analysis was conducted on the DL model, separately for each training fold. To determine the overall feature importance, the mean and standard deviation of the calculated permutation feature importance were computed by averaging the importance values across folds. Figure 2 illustrates the mean and standard deviation of the permutation feature importance for all features.

Our findings reveal that the feature with the highest importance was BD_2cc_, followed by BD_5cc_ and BP_ICRU_. Additionally, the features with the highest importance within each group were age, TNM category, number of CCRT cycles, and BD_2cc_ for the patient, tumor, treatment, and dose feature groups, respectively. 

We identified the top five features with high importance as BD_2cc_, BD_5cc_, BP_ICRU_, TNM category, and tumor size. We made the decision to select tumor size instead of FIGO stage as a feature, considering the possibility of redundancy between FIGO stage and TNM category. This decision was based on the scientific rationale that tumor size provides valuable and distinct information for the prediction model [37].

### 3.4. Deep Learning Models

**Figure 3:**
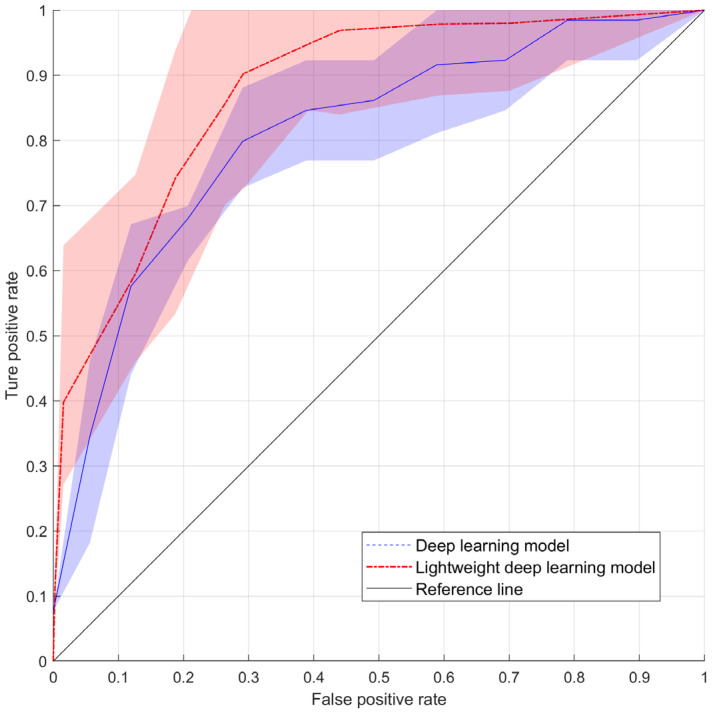
The area under the receiver operating curve comparison across 5-fold cross-validation with the reference line.

The prediction performance of the DL model for late bladder toxicity was evaluated using the voting method. Table 4 summarizes the prediction performances of the DL models and the lightweight DL models trained using the five different training folds. The means and standard deviations of prediction performance for accuracy, precision, recall, and F1-score differed between the deep learning model and the lightweight deep learning model. For the deep learning model, the values were as follows: accuracy (0.77 ± 0.06), precision (0.68 ± 0.10), recall (0.41 ± 0.12), and F1-score (0.49 ± 0.05). On the other hand, the lightweight deep learning model had different values: accuracy (0.92 ± 0.03), precision (0.97 ± 0.03), recall (0.86 ± 0.07), and F1-score (0.90 ± 0.04). In the case of the AUROC, the deep learning model achieved a value of 0.81 ± 0.04, while the lightweight deep learning model achieved a value of 0.94 ± 0.03 (Figure 3).

Finally, both the DL model and the lightweight deep learning model were evaluated using the voting method with a threshold of 3. For the DL model, the evaluation metrics were as follows: Accuracy (0.91), Precision (0.85), Recall (0.69), F1-score (0.76), and AUROC (0.81). For the lightweight deep learning model, the evaluation metrics were as follows: Accuracy (0.93), Precision (0.94), Recall (0.88), F1-score (0.90), and AUROC (0.91). 

However, because the AUROC could not be calculated from the labels predicted by each of the five models, the average of the AUROCs of all the models was considered as the overall AUROC value.

## 4. Discussion

This study aimed to (1) compare the ability of multivariable logistic regression and DL models to identify those patients who, having received radiation therapy, are at risk of bladder radiation toxicity, and (2) interpret the results of DL models to understand the significance of input features. To the best of our knowledge, no previous study has attempted an interpretation to ensure the reliability of a DL model in predicting the occurrence of late bladder toxicity.

Several statistical methods and DL models are available for predicting clinical outcomes, including radiation toxicity. In many instances, DL models can effectively find near-optimal solutions for nonconvex optimization problems using gradient methods and nonlinear activation functions. However, statistical methods can also achieve high accuracy and reliability in specific cases. Despite the rapid development of DL models and their relatively high performance, their clinical utility is still a topic of controversy due to concerns about their reliability, particularly in terms of how clinicians interpret the results and features.

In this study, we utilized an MLP with relatively low model complexity to enhance the interpretability of deep learning models. The performance of the MLP relies on the configuration of the hidden layer, and Muhammad et al. [42] suggested that the optimal configuration for the hidden layer is three. Furthermore, experimental findings demonstrated that having fewer than three hidden layers directly impacts the network’s accuracy, while having more than three hidden layers increases the time complexity without a proportional improvement in accuracy. Determining the appropriate number of neurons in the hidden layer is still a topic of debate, and the design of the model structure should be customized to the specific problem and available computational resources while also considering the general MLP model design standard: a structure in which the number of neurons in the hidden layer continuously decreases. Therefore, we selected the top five important features based on the results of the permutation feature importance analysis and designed the lightweight DL model, taking into account the general design standard. This approach not only improves interpretability but also offers advantages associated with the general design standard of MLP models. For instance, the continuous reduction of neurons in the hidden layer helps prevent overfitting and allows for efficient learning of complex patterns while avoiding excessive computational complexity.

Considering the experiment on our dataset, the DL model proved superior compared with the statistical model. To address the class-imbalance problem in the DL method, we adopted the SMOTE oversampling technique. Mylona et al. [18] suggested that variations in oversampling techniques, including SMOTE, increase the prediction performance of classifiers. Accordingly, our results showed that when SMOTE was not applied to the training process, the mean and standard deviation of the AUROC value on the test set were reduced to 0.52 ± 0.13. 

**Figure 4:**
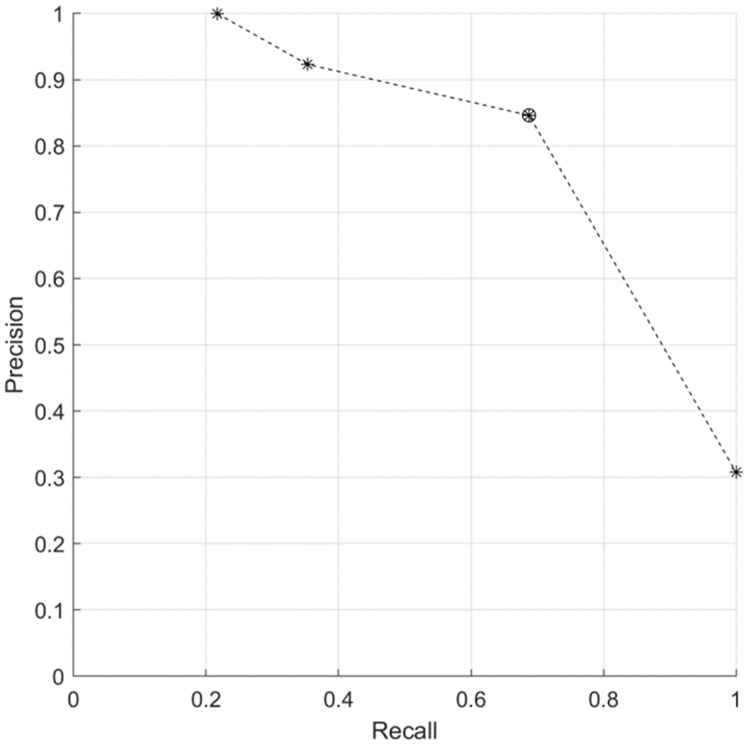
Precision–recall curve depending on the voting method threshold value. The asterisk in a circle indicate a threshold of 3.

The precision and recall of the DL model exhibited a tradeoff relationship depending on the value of the threshold used for voting, as shown in Figure 4. For the prediction of radiation-induced bladder toxicity, both precision and recall are crucial metrics for assessing the performance of a prediction model because they reflect the costs and benefits associated with false positives and false negatives. High precision is essential to minimize unnecessary interventions and potential harm for patients who are not at risk. However, a high recall is necessary to ensure that all patients at risk of developing bladder toxicity are detected and appropriate measures are taken to mitigate the associated risks. Therefore, achieving a balance between precision and recall is necessary to optimize the performance of the prediction model for clinical applications. At a threshold of 3, the precision and recall of the voting methods are indicated by markers placed inside circles denoted by asterisks.

Permutation feature importance is a model-agnostic technique that provides a comprehensive and computationally efficient assessment of feature importance by considering feature interactions and avoiding the bias caused by collinear or redundant features. In this study, permutation feature importance is utilized to calculate the relative importance of features while also employing a feature reduction technique like principal component analysis [42,43,44]. The importance of permutation features can be sensitive to the choice of the metric used to evaluate the model‘s performance (see Equation (7)), leading to different feature rankings. Our findings emphasize the importance of specific features, such as BD_2cc_, BD_5cc_, and BP_ICRU_ for the accurate prediction of late bladder toxicity occurrence, which is also consistent with the prognostic factor for urinary toxicity suggested in previous studies, such as BD_2cc_ in [5]. Our results provide insights that could facilitate the development of more effective and personalized treatment strategies for patients undergoing radiation therapy.

Our study has certain limitations that need to be considered. Firstly, our analysis was based solely on structured data (patient and treatment dose features), which may not fully capture the complexity of bladder toxicity. Recent studies have demonstrated the potential of more sophisticated DL models that incorporate 3D dose distributions, medical images, contours, etc. to predict clinical outcomes [45,46]. Radiomics, which involves extracting quantitative features from medical images, can provide additional information about tumor characteristics and treatment response. Integrating radiomics data alongside patient data and dose volumetric parameters has shown improvements in the prediction of various types of toxicities, with potential enhancements in AUC values ranging from 0.11 to 0.16 [13]. Therefore, in future research, we aim to explore the integration of radiomics data into our DL models to enhance the prediction of late bladder toxicity. By incorporating this additional information, we anticipate improved performance and a better understanding of the underlying factors contributing to toxicity with advanced interpretation techniques.

Secondly, although the DL model outperformed the statistical model in our dataset, it is important to note that the performance of DL models can vary depending on the specific dataset and problem domain. Further validation using larger and more diverse datasets through multi-institutional studies is required to confirm the generalizability of our findings.

Thirdly, interpreting DL models remains a challenging task, especially concerning feature importance. Permutation feature analysis is a useful technique for assessing the importance of features in DL models. However, it should be noted that this method has limitations. It tends to assign higher importance to continuous variables and can produce different feature rankings depending on the choice of evaluation metric. Additionally, applying this technique to 3D input is challenging. While permutation feature analysis provides valuable insights, it needs to be supplemented with other interpretive techniques to gain a more comprehensive understanding of the behavior and functional importance of DL models. Therefore, as a further study, it is necessary to apply various analysis methods, such as LIME and its variants, input gradient-based methods, CAM and its variants, etc., to the DL model to ensure the reliability of models with relatively higher performance [47,48].

Furthermore, in order to address the limitation of a small sample size, we established an extra-validation set consisting of 17 individuals. Utilizing a lightweight DL model, we conducted predictions for late bladder toxicity. The results revealed an accuracy of 0.81%, a precision of 0.99%, a recall of 0.61%, an F1-score of 0.92, and an AUROC of 0.93. These performance metrics indicate that the lightweight DL model had limited accuracy in forecasting late bladder toxicity. While the precision was high, indicating few false positives, the recall was relatively low, meaning it missed many true positive cases. 

It is crucial to exercise caution when interpreting these findings due to the relatively small size of the extra-validation set. The restricted sample may not adequately encompass the full range of late bladder toxicity in radiation therapy. For instance, the longest observed duration of late bladder toxicity in our institution was 107.7 months from the initiation of external beam radiation therapy (EBRT) to the occurrence of late toxicity. As a result, the performance metrics on the extra-validation set obtained may not truly reflect the predictive capabilities of the DL model. Thus, future research should strive to incorporate a larger patient cohort from multiple institutions to validate and enhance the predictive capabilities of DL models in accurately anticipating late bladder toxicity.

Overall, this study contributes to the ongoing discussion on the clinical utility of DL models in predicting radiation toxicity and emphasizes the importance of interpretability to enhance the reliability and practical applicability of these models. By addressing the limitations and conducting further research, we can advance the field and ultimately improve patient outcomes in radiation therapy.

## 5. Conclusions

In this study, we compared the performance of logistic regression and DL models for the prediction of late bladder toxicity in patients with cervical cancer. Logistic regression did not show acceptable performances, whereas the lightweight DL model achieved an accuracy of 0.92 ± 0.03 and an AUROC of 0.91 ± 0.03. Moreover, the permutation feature importance analysis identified BD_2cc_ as the most important feature for risk prediction. 

## Figures and Tables

**Table 1:** The architecture of the late bladder toxicity prediction model.

Layer Type	In Features	Out Features	Bias	Batch Normalization	Activation Function
Fully connected layer	16	36	FALSE	TRUE	* Leaky ReLU
Fully connected layer	36	72	FALSE	TRUE	Leaky ReLU
Fully connected layer	72	144	FALSE	TRUE	Leaky ReLU
Fully connected layer	144	72	FALSE	TRUE	Leaky ReLU
Fully connected layer	72	36	FALSE	TRUE	Leaky ReLU
Fully connected layer	36	1	FALSE	TRUE	Sigmoid

* Leaky ReLU: Leaky rectified linear unit.

**Table 2:** Baseline patients’ characteristics (n = 281).

Variable			Total (N = 281)		Training Set (N = 199)		Test Set (N = 82)		*p*-Value
AGE		mean ± std	62.1	±14.0	63.4	±13.4	61.5	±14.2	0.2920 ^(3)^
Pathology	1;	squamous	224	(79.7%)	63	(76.8%)	161	(80.9%)	0.4708 ^(1)^
	2;	adenoca	23	(8.2%)	6	(7.3%)	17	(8.5%)	
	3;	adenosquamous	10	(3.6%)	5	(6.1%)	5	(2.5%)	
	4;	Other	24	(8.5%)	8	(9.8%)	16	(8.0%)	
FIGO stage	1;	Ia1, Ia2, Ib1, Ib2	53	(18.9%)	17	(20.7%)	36	(18.1%)	0.7285 ^(1)^
	2;	IIa1, IIa2, Iib	161	(57.3%)	44	(53.7%)	117	(58.8%)	
	3;	IIIa, IIIb, Iva, Ivb	67	(23.8%)	21	(25.6%)	46	(23.1%)	
TNM stage	1;	T1a1, T1a2, T1b1, T1b2	56	(19.9%)	17	(20.7%)	39	(19.6%)	0.9752 ^(1)^
	2;	T2a1, T2a2, T2b	184	(65.5%)	53	(64.6%)	131	(65.8%)	
	3;	T3a, T3b, T4	41	(14.6%)	12	(14.6%)	29	(14.6%)	
CCRT	0;	RT alone	22	(7.8%)	4	(4.9%)	18	(9.0%)	0.2372 ^(1)^
	1;	CCRT	259	(92.2%)	78	(95.1%)	181	(91.0%)	
Concurrent chemotherapy regimen	1;	cisplatin	231	(89.2%)	72	(92.3%)	159	(87.8%)	0.7583 ^(2)^
(CCRT 259 case)	2;	5FU + cisplatin	3	(1.2%)	0	(0.0%)	3	(1.7%)	
	3;	carboplatin	19	(7.3%)	5	(6.4%)	14	(7.7%)	
	4;	other	6	(2.3%)	1	(1.3%)	5	(2.8%)	
Number of concurrent chemotherapy cycle	0;	Cycle 3 or less	26	(10.0%)	7	(9.0%)	19	(10.5%)	0.7083 ^(1)^
(CCRT 259 case)	1;	Cycle 3 or more	233	(90.0%)	71	(91.0%)	162	(89.5%)	
Adjuvant chemotherapy	0;	No	260	(92.5%)	79	(96.3%)	181	(91.0%)	0.1185 ^(1)^
	1;	Yes	21	(7.5%)	3	(3.7%)	18	(9.0%)	
Tumor size (cm)(MRI axial)		median (min–max)	4.2	(1.3–10)	4.3	(2.3–8.5)	4.2	(1.3–10)	0.6955 ^(3)^
EBRT total dose EQD2(3) (Gy)		median (min–max)	48.4	(20–73.1)	48.4	(43.2–68.3)	48.4	(20–73.1)	0.2765 ^(3)^
GTV D100 (cGy)		median (min–max)	570.5	(112.3–1336)	552.4	(194–1187.8)	584.2	(112.3–1336)	0.1644 ^(3)^
BPICRU EQD2(3) (Gy)		median (min–max)	23.5	(0–93.2)	26.9	(0–93.2)	22.5	(0–90.7)	0.1775 ^(3)^
BD0.1cc EQD2(3) (Gy)		median (min–max)	58.3	(12.6–202.4)	59.7	(25–174)	57.6	(12.6–202.4)	0.2659 ^(3)^
BD1cc EQD2(3) (Gy)		median (min–max)	46.1	(10–141.7)	48.9	(20.5–111.1)	45.6	(10–141.7)	0.1906 ^(3)^
BD2cc EQD2(3) (Gy)		median (min–max)	41.3	(6.3–120.5)	43.6	(9.8–97.8)	39.8	(6.3–120.5)	0.2492 ^(3)^
BD5cc EQD2(3) (Gy)		median (min–max)	33.5	(1.3–91.5)	35.2	(3.8–78.9)	33	(1.3–91.5)	0.1944 ^(3)^

(1) Chi-square test. (2) Fisher’s exact test. (3) *t*-test.

**Table 3:** Results of univariable and multivariable logistic regression analysis for late bladder toxicity prediction.

Variable			Univariable Analysis		Multivariable Analysis	
			OR (95% CI)	*p*-Value	OR (95% CI)	*p*-Value
AGE			0.997 (0.975–1.019)	0.803		
Pathology	1;	squamous	1 (ref)			
	2;	adenoca	0.769 (0.238–2.483)	0.661		
	3;	adenosquamous	1.667 (0.270–10.303)	0.583		
	4;	Other	0.357 (0.078–1.634)	0.185		
FIGO stage	1;	Ia1, Ia2, Ib1, Ib2	1 (ref)			
	2;	IIa1, IIa2, Iib	0.937 (0.406–2.164)	0.879		
	3;	IIIa, IIIb, Iva, Ivb	1.024 (0.388–2.706)	0.962		
TNM stage	1;	T1a1, T1a2, T1b1, T1b2	1 (ref)			
	2;	T2a1, T2a2, T2b	0.820 (0.375–1.794)	0.620		
	3;	T3a, T3b, T4	0.716 (0.241–2.127)	0.548		
CCRT	0;	RT alone	1 (ref)			
	1;	CCRT	1.336 (0.42–4.252)	0.624		
Concurrent chemotherapy regimen	1;	cisplatin	1 (ref)			
(CCRT 181 case)	2;	5FU + cisplatin	5.066 (0.448–57.267)	0.190		
	3;	carboplatin	0.422 (0.091–1.962)	0.271		
	4;	other	0.633 (0.069–5.821)	0.687		
Concurrent chemotherapy cycle	0;	Cycle 3 or less	1 (ref)		1 (ref)	
(CCRT 181 case)	1;	Cycle 3 or more	0.481 (0.181–1.277)	0.142	0.440 (0.162–1.194)	0.107
Adjuvant chemotherapy	0;	No	1 (ref)			
	1;	Yes	1.385 (0.493–3.897)	0.537		
Tumor size (cm)(MRI axial)			1.011 (0.828–1.234)	0.918		
EBRT total dose EQD2(3) (Gy)			1.006 (0.951–1.065)	0.831		
GTV D100 (cGy)			1.001 (0.999–1.003)	0.186	1.002 (1.000–1.004)	0.136
BPICRU EQD2(3) (Gy)			1.005 (0.985–1.025)	0.651		
BD0.1cc EQD2(3) (Gy)			1.008 (0.995–1.020)	0.232		
BD1cc EQD2(3) (Gy)			1.012 (0.993–1.031)	0.213		
BD2cc EQD2(3) (Gy)			1.016 (0.996–1.036)	0.114	1.047 (0.939–1.168)	0.406
BD5cc EQD2(3) (Gy)			1.018 (0.993–1.043)	0.163	0.961 (0.841–1.100)	0.566

**Table 4:** Performance comparison for predicting late bladder toxicity of a statistical model and DL models by five different folds for the test data.

Model	Accuracy	Precision	Recall	F1-Score	* AUROC
Statistical model	0.85	0.08	0.5	0.14	0.43
Deep learning model: fold 1	0.78	0.62	0.38	0.47	0.76
Deep learning model: fold 2	0.73	0.85	0.35	0.50	0.86
Deep learning model: fold 3	0.76	0.69	0.36	0.47	0.84
Deep learning model: fold 4	0.70	0.69	0.30	0.42	0.83
Deep learning model: fold 5	0.88	0.54	0.64	0.58	0.77
Deep-learning model: voting method (threshold = 3)	0.91	0.85	0.69	0.76	0.81
Leightweight deep learning model: fold 1	0.93	0.99	0.83	0.92	0.93
Leightweight deep learning model: fold 2	0.88	0.94	0.81	0.87	0.88
Leightweight deep learning model: fold 3	0.90	0.99	0.81	0.89	0.90
Leightweight deep learning model: fold 4	0.97	0.94	0.99	0.98	0.97
Leightweight deep learning model: fold 5	0.91	0.98	0.85	0.86	0.89
Deep-learning model: voting method (threshold = 3)	0.93	0.94	0.88	0.90	0.91

* AUROC: area under the receiver operating characteristic curve.

## Data Availability

Some or all datasets generated during and/or analyzed during the current study are not publicly available but are available from the corresponding author on reasonable request.
